# Nurses' self‐efficacy and well‐being at work amid the COVID‐19 pandemic: A mixed‐methods study

**DOI:** 10.1002/nop2.1752

**Published:** 2023-04-07

**Authors:** Nahed Alquwez

**Affiliations:** ^1^ Department of Nursing, College of Applied Medical Sciences Shaqra University Al Dawadmi Saudi Arabia

**Keywords:** COVID‐19 pandemic, mixed‐methods, nurses, self‐efficacy, well‐being

## Abstract

**Aims:**

To explore the factors associated with the nurses' well‐being at work.

**Design:**

A sequential explanatory mixed‐methods design.

**Methods:**

The quantitative part of the study included a conveniently sampled 271 nurses employed in healthcare facilities in the Riyadh region of Saudi Arabia. A purposive sample of 21 nurses were interviewed in the qualitative part of the study. Data collection was performed from May to August 2021. This article followed the STROBE checklist.

**Results:**

Nurses working in private hospitals reported higher level of self‐efficacy than nurses in public hospitals. Being a Filipino, working in private hospital, and having higher self‐efficacy were associated with better well‐being at work among nurses during the pandemic. The thematic analysis revealed four important themes in understanding their well‐being at work: safe work environment, ensuring staff nurses' health, leadership support, and solidarity in the workplace.

## 1 INTRODUCTION

The current pandemic has caused extraordinary and long‐lasting work demands on nurses. Nurses spend more time accomplishing various responsibilities, including providing nursing care to patients, attending to the needs of the patient's family, and performing administrative functions. Nurses work tirelessly during their shifts, enduring faulty work environment conditions, inadequate protective resources, and situations that challenge them emotionally and mentally (Alsolais et al., 2021; WHO, 2020a). A previous study reported that nurses' professional quality of life is affected by their work experiences and conditions during this pandemic (Inocian et al., 2021). These situations for nurses have placed their well‐being at work at risk. Nurses' self‐efficacy to perform and execute at different levels in response to care for COVID‐19 has likely decreased as compared to non‐pandemic situations. Thus, they are putting their well‐being at work at risk. Taking care of nurses' well‐being directly affects their ability to fully serve their patients because negative well‐being in the workplace brings consequences in areas such as performance, absenteeism, and staff turnover (WHO, 2020a). Although well‐being is considered important for nurses, the relationship between self‐efficacy among nurses during challenging situations, such as the COVID‐19 pandemic, has rarely been assessed. The underlying mechanism of this relationship, especially during a pandemic, needs to be understood. Addressing the self‐efficacy and overall well‐being at work requires understanding the nature and extent of the effects of pandemics to ensure that effective interventions can be used to guarantee the optimum levels of nurses' well‐being at work. Having optimum well‐being at work among nurses and other healthcare workers is critical to ensuring the provision of high‐quality and safe care to patients. Therefore, this study examined nurses' perceived self‐efficacy and well‐being at work during the pandemic. It explored the factors associated with the nurses' well‐being at work using quantitative and qualitative methodologies.

### 1.1 Background

The COVID‐19 disease is a communicable respiratory infection that has affected all aspects of life and has caused millions of morbidities and mortalities around the globe (Centers for Disease Control and Prevention, 2020). Different governments have executed different global actions to control the spread of the virus and mitigate its health, social and economic effects (Ehrenberg et al., 2021). Countries have rolled out vaccination programs with the ultimate goal of developing herd immunity despite many challenges related to the unbalanced supply and demand of the COVID‐19 vaccine and vaccine hesitancy among the public (Alshehry et al., 2022). In Saudi Arabia, the government has imposed country‐specific measures parallel with the WHO standards in combating outbreaks, such as effective testing, prevention, control, and infection treatment (Albaqawi et al., 2020). The country has also been very aggressive in its vaccination program, with more than 45% of its population fully vaccinated. However, the demand for healthcare to attend to the needs of COVID‐19 patients remains a challenge for most countries, and hospitals are continuously pressured to cope with these increasing demands. Hospitals face numerous challenges in responding to this pandemic, including the healthcare workforce shortage, the inadequacy of medical and personal protective equipment supplies, and logistical matters (Willan et al., 2020). With the increasing admission rates in the hospitals, even well‐staffed hospitals experience increased infection risks, fear, stress, anxiety, burnout, and emotional strain on their healthcare workers, including nurses.

Nurses are frontline healthcare professionals who perform a significant part in COVID‐19 prevention, infection control, and management (Huang et al., 2020). The tasks and responsibilities of nurses are crucial because of their consistent contact with patients during the care process (Albougami et al., 2020), making them at high risk of getting infected with COVID‐19. Moreover, previous studies have reported various negative experiences of nurses while assuming their important roles in the fight against the COVID‐19 pandemic, such as emotional and mental health problems, physical burden, challenging work environment and situations, excessive workload, and unmet needs (Ahmadidarrehsima et al., 2022; Alquwez et al., 2022). The negative experiences of nurses during this pandemic have had a direct or indirect effect on their overall health and well‐being.

Well‐being at work is significant and needs to be emphasized amid the exhausting work experiences of nurses during the pandemic. Taking care of nurses' well‐being significantly affects their ability to fully serve their patients (Alshehry et al., 2019; Cruz, 2016). However, negative well‐being in the workplace brings consequences such as poor performance, absenteeism, high staff turnover, and reduced work‐life balance (Albougami et al., 2020; WHO, 2020b). According to Biggio and Cortese (2013), well‐being at work corresponds to all features of working life and how a person feels about their employment, work organization, and work environment. The International Labor Organization (2009) argued that well‐being at work is an essential aspect of defining an organization's success, productivity levels, and overall well‐being. Thus, nurses' health and well‐being in the workplace must be looked after and guaranteed because they are important in providing the best care and in achieving excellent patient outcomes amid the pandemic. However, while the significance of well‐being at work is widely recognized, nurses' well‐being does not appear to be a priority in the clinical setting during health crises, such as the COVID‐19 pandemic.

Nurses may feel that they are losing their capacity to control their situation and the clinical environment during the pandemic, which may affect their behaviour, way of thinking, and future events. This situation indicates low self‐efficacy (Bandura, 2010). According to Bandura (2010), self‐efficacy suggests “an overall self‐confidence that an individual responds to different environmental challenges or faces new things.” That is, self‐efficacy permits an individual to handle a crisis. Empirical data noted that self‐efficacy is linked to anxiety, depression, and stress (Tahmassian & Jalali Moghadam, 2011; Yao et al., 2018). Research has shown that higher self‐efficacy supported individuals' mental well‐being and positive coping with stress (Shakespeare‐Finch et al., 2014; Yao et al., 2018). Therefore, exploring nurses' self‐efficacy during the COVID‐19 pandemic is necessary to understand how their confidence in responding and coping with this difficult situation were affected.

## 2 METHODS

A sequential explanatory mixed‐methods study was performed to explore the nurses' self‐efficacy and well‐being at work amid the COVID‐19 pandemic. A quantitative study was first conducted to examine the nurses' self‐efficacy and well‐being at work and the factors influencing their well‐being at work. The quantitative data are utilized to explain the primary question (Tashakkori & Teddlie, 1998). Subsequently, a qualitative inquiry was performed to provide more clarity on the findings of the quantitative inquiry. The qualitative data are embedded to explain the related predictors and variables of the quantitative data (Tashakkori & Teddlie, 1998). The study aims to explore the nurses' self‐efficacy and well‐being at work amid the COVID‐19 pandemic, which is addressed first by quantitative research. According to Morgan (1998), in choosing a primary method in a sequential study, the researcher should consider the method suited to answer the objective or purpose of the study. The researcher uses validating qualitative research to expand and validate the quantitative findings (Creswell et al., 2003). The qualitative data supplement the quantitative data since the qualitative data rigorously explain the finding that a quantitative data set cannot explain (Tashakkori & Teddlie, 1998). In addition, qualitative data provide themes used by the researcher to validate and explain the quantitative result (Creswell et al., 2003). Furthermore, Tashakkori and Teddlie (1998) mentioned that the emotional and psychological aspect of a phenomenon that a quantitative results survey cannot explain is supported by the qualitative result. Thus, the qualitative data further explain the nurses' self‐efficacy and well‐being at work items amid the COVID‐19 pandemic. This article followed the STROBE checklist in its reporting (Supplementary File 1).

### 2.1 Participants

The quantitative part of the study included a conveniently sampled 271 nurses employed in healthcare facilities in the Riyadh region of Saudi Arabia. The inclusion criteria were as follows: nurse in Saudi Arabia, employed in any hospitals in the Riyadh region during the pandemic, full‐time employee, and with at least six months of employment in the hospital. Table 1 reflects the demographic characteristics of the respondents. For the qualitative part of the study, a purposive sample of 21 nurses participated in the study. These nurses were part of the sample of the quantitative phase of the study. Hence, in addition to the inclusion criteria in the quantitative part, the item, those who expressed their willingness to be interviewed, was added to the criteria for inclusion. The demographic characteristics of the participants in the qualitative part can be found in Supplementary File 2.

**TABLE 1:** Demographic characteristics of the respondents (*n* = 271).

Variable	Mean (SD)	Range
Age	34.08 (6.54)	21.00–55.00
Years of experience	11.20 (5.97)	1.00–31.00
Gender	*n*	%
Male	85	31.4
Female	186	68.6
Marital status		
Single	119	43.9
Married	152	56.1
Nationality		
Saudi	55	20.3
Filipino	133	49.1
Indian	83	30.6
Religion		
Islam	75	27.7
Christianity	180	66.4
Hinduism	16	5.9
Education		
Diploma in nursing	32	11.8
BSN	205	75.6
Graduate program	34	12.5
Hospital		
Public hospital	184	67.9
Private hospital	87	32.1
Bed capacity		
100 beds and below	71	26.2
101 to 200 beds	141	52.0
201 to 300 beds	22	8.1
301 beds and above	37	13.7
Clinical area		
Emergency Department	53	19.6
Outpatient Department	23	8.5
Medical Department	29	10.7
Surgical Department	22	8.1
ICUs	44	16.2
Operating Rooms	29	10.7
Obstetric Department	12	4.4
Artificial Kidney Unit	11	4.1
Nursing Administration	31	11.4
Oncology	17	6.3
Provided direct nursing care to COVID‐19 patients		
No	79	29.2
Yes	192	70.8
Previous COVID‐19 infection		
No	192	70.8
Yes	79	29.2
COVID‐19 vaccination status		
1st Dose	54	19.9
Completed	217	80.1

### 2.2 Instrument

#### 2.2.1 Quantitative part

Data were collected using an online survey. The online survey comprised of three sections. Section one contained questions pertaining to the respondents' demographic and work‐related variables. The data collected include (1) age, (2) sex, (3) marital status, (4) nationality, (5) religion, and (6) highest educational attainment. For the work‐related variables, the following information was obtained: (1) hospital type, (2) bed capacity, (3) practice area, (4) years of experience, (5) experience providing care to patients with COVID‐19 infection, (6) previous COVID‐19 infection, and (7) COVID‐19 vaccination status.

Section 2 used the “General Self‐Efficacy Scale” (GSE). The GSE assesses the “general sense of perceived self‐efficacy with the aim in mind to predict coping with daily hassles as well as adaptation after experiencing all kinds of stressful life events”. The GSE has 10 items with four response options: “not at all true, hardly true, moderately true, and exactly true.” The items of the scale were designed to reflect successful coping with stressful life events and infer an internally stable attribution of success. To frame the items in the scale in relation to the COVID‐19 crisis experience of the nurse, the following question was included before the scale: “Based on your experience in the clinical settings during the COVID‐19 crisis, kindly provide your most honest response on the following items using the following options.” The scoring of the tool can be obtained by summing up the individual scores of each item, which could range from 10 to 40. A higher GSE score indicates a higher level of self‐efficacy. The GSE is a valid and reliable measure with Cronbach's alpha reported in various studies ranging from 0.76–0.90 (Schwarzer & Jerusalem, 1995). Previous studies employed the GSE to measure the nurses' self‐efficacy during the COVID‐19 pandemic; hence, its use in the present study (e.g., Peñacoba et al., 2021; Sharour et al., 2022; Xiong et al., 2020).

Section 3 presented the “Nurses' Well‐being at Work Scale” (NWB) (Päätalo & Kyngäs, 2016), which was created to assess the well‐being of nurses in the hospital setting. The NWB has 67 items divided into 12 factors: “patients' experience of high‐quality care (8 items), assistance and support among nurses (9 items), nurses' togetherness and collaboration (10 items), satisfying practical organization of work (7 items), challenging and meaningful work (6 items), freedom to express diverse feelings in a work community (4 items), well‐conducted everyday nursing (5 items), status related to the work itself (7 items), fair and supportive leadership (3 items), opportunities for professional development (3 items), fluent communication with other professionals (3 items), and being together with other nurses in an informal way (2).” The NWB measures the significance of each proposition to nurses. To frame the questions in relation to the COVID‐19 crisis experience of the nurse, the following question was included before the scale: “Based on your experience in the clinical setting during the COVID‐19 pandemic, kindly evaluate the extent of the importance of the following propositions to your well‐being at work” The scale uses a five‐point Likert scale (1 “to a very small extent” to 5 “to a very great extent”). Mean scores were calculated with a higher mean signifying better well‐being at work. The scale is a valid and reliable tool with Cronbach's alpha ranging from 0.66 to 0.91 (Päätalo & Kyngäs, 2016).

#### 2.2.2 Qualitative part

Data collection for this part was performed using online, semi‐structured interviews for a deeper exploration of the nurses' self‐efficacy and well‐being at work during their experience in the pandemic. The interviews, which were done using the Zoom application, were conducted online because of the restrictions of gathering imposed in the settings during the data collection period. The primary question in the interview was “How can you describe your well‐being at work during the pandemic?” Follow‐up questions stem from the answer to the primary question to further explore their self‐efficacy and well‐being.

### 2.3 Data collection

#### 2.3.1 Quantitative part

The data collection was conducted using Survey Monkey from May to August 2021. A study recruitment message containing basic information of the study and the link to the online survey was forwarded to potential nurses using various social media applications, such as WhatsApp, Twitter, Facebook, Snapchat, and Instagram. A similar message was posted in social media groups of nurses in the particular area of interest of the study. interested participants were directed to click the link to the survey and were directed to the study information section and the electronic informed consent. Those who clicked “agree” to the informed consent continued to the survey, while those who clicked “do not agree” were automatically brought to the end of the survey. Follow‐up messages were sent every week until the last day of the data collection period.

#### 2.3.2 Qualitative part

The end of the online survey contained an invitation for an interview, as well as the contact information of the researcher. This section also contained information on the qualitative part of the study. The nurses who wanted to participate in the interview contacted the researcher. The researcher arranged the date and time of the online interview and provided the Zoom link to the participant. The interviews lasted for 45–60 min. The interviews were conducted using the Arabic language and were recorded using the Zoom record feature. The data collection was conducted consecutively until data saturation was achieved. After the data were collected, it was transcribed by the researcher, the researcher asked for the help of two qualitative researchers. Each transcript file was manually coded by the researcher and the other two qualitative researchers. After the coding, the researcher and the two qualitative researchers compare their code and if there are discrepancies, they reread the data and re‐code it. Then, the codes were extracted and themes were developed. After the themes were developed, an external qualitative researcher evaluated the data, code, and themes to make a rigorous finding since it provided more objective and unbiased looks at the findings of the study. Lastly, the final result of the qualitative data is written.

### 2.4 Data analyses

#### 2.4.1 Quantitative part

Using SPSS version 22.0, descriptive statistical analyses were performed for the demographic and work‐related variables, self‐efficacy, and well‐being at work. Multiple regression analysis was conducted to determine the factors associated with well‐being at work with demographic and work‐related variables and self‐efficacy as predictor variables. Non‐continuous data were dummy coded before analyses. Pearson's correlations were determined to test the associations that existed between the dimensions of self‐efficacy and well‐being at work. P‐values less than 0.05 were considered significant. 95% CI was reported accordingly.

#### 2.4.2 Qualitative part

A thematic approach to qualitative data analysis was followed in the data analysis for this part of the paper. The thematic analysis followed several steps, including “familiarization with the data, coding the data, determining patterns among the codes and beginning the development of the themes, reviewing the themes by going back to the data and comparing the themes against the data, defining and naming the themes, and writing the thematic analysis” (Nowell et al., 2017).

### 2.5 Data integration

Data integration was employed because the participants of the study were selected from those who answered the quantitative part (Dowding, 2013). The quantitative and qualitative data were collected sequentially and a contagious narrative approach was used to integrate the results (Stange, 2006). The qualitative and qualitative parts were analysed separately and then a single report was formulated. The quantitative part was analysed using descriptive and inferential statistics, and themes were developed from the qualitative part.

### 2.6 Rigor/Trustworthiness

Effort was made to ensure rigor in the qualitative part of the study. To address the researcher's prejudices, ongoing testing of pre‐research assumptions was conducted during the data interpretation by performing comparisons of the assumptions and the findings. This process guaranteed that the data were interpreted from the participants' point of view and not from the researcher's perspective. Moreover, the transcripts were reviewed by the researcher and two external evaluators. To ensure credibility, the interpretations of the interviews were presented to the participants for validation. They were asked to evaluate whether the findings were reflective of their experiences. They were asked whether the interpreted statements reflected what they meant when they were interviewed.

## 3 RESULTS

### 3.1 Quantitative result

#### 3.1.1 Self‐efficacy among nurses

Table 2 reflects the descriptive findings on the self‐efficacy among nurses. The overall mean score of the nurses in the GSE was 32.18 (SD = 4.49) with scores ranging from 18 to 40. This finding indicates a high level of self‐efficacy among the nurses during the pandemic. The nurses gave the highest rating on the item “I can solve most problems if I invest the necessary effort” (*M* = 3.41, SD = 0.60), followed by “I can always manage to solve difficult problems if I try hard enough” (*M* = 3.34, SD = 0.67). The item “If someone opposes me, I can find the means and ways to get what I want” received the lowest rating (*M* = 2.80, SD = 0.90).

**TABLE 2:** Descriptive statistics results of the nurses' self‐efficacy (*n* = 271).

Variables	Minimum	Maximum	Mean	SD
1. I can always manage to solve difficult problems if I try hard enough	1.00	4.00	3.34	0.67
2. If someone opposes me, I can find the means and ways to get what I want	1.00	4.00	2.80	0.90
3. It is easy for me to stick to my aims and accomplish my goals	1.00	4.00	3.14	0.74
4. I am confident that I could deal efficiently with unexpected events	1.00	4.00	3.25	0.73
5. Thanks to my resourcefulness, I know how to handle unforeseen situations	1.00	4.00	3.25	0.64
6. I can solve most problems if I invest the necessary effort	2.00	4.00	3.41	0.60
7. I can remain calm when facing difficulties because I can rely on my coping abilities	1.00	4.00	3.29	0.65
8. When I am confronted with a problem, I can usually find several solutions	1.00	4.00	3.21	0.64
9. If I am in trouble, I can usually think of a solution	1.00	4.00	3.27	0.71
10. I can usually handle whatever comes my way	1.00	4.00	3.24	0.69
Self‐efficacy mean total score	18.00	40.00	32.18	4.49

*Note*: Possible score range = 10–40.

Among the demographic variables, only the type of hospital was found to have a significant relationship with nurses' self‐efficacy. Specifically, nurses working in private hospitals (*M* = 33.09, SD = 4.13) reported significantly higher level of self‐efficacy than nurses in public hospitals (*M* = 31.75, SD = 4.59, *t* = −2.31, *p* = 0.021).

#### 3.1.2 Nurses' well‐being at work and its associated factors

The descriptive analyses results on the well‐being at work are shown in Table 3. The overall mean score of the respondents was 255.08 (SD = 45.24) from a possible range of scores of 67–335. This indicates a moderate level of well‐being at work among nurses during the pandemic. Among the dimensions of the well‐being at work, “Assistance and support among nurses” received the highest mean (*M* = 4.20, SD = 0.63), followed by “Nurses' togetherness and collaboration” (*M* = 4.01, SD = 0.78), and “Patients' experience of high‐quality care” (*M* = 4.00, SD = 0.57). The dimensions “Being together with colleagues in an informal way” (*M* = 3.37, SD = 1.05) and “Satisfying practical organization of work” (*M* = 3.30, SD = 0.97) received the lowest mean scores.

**TABLE 3:** Results of the descriptive analyses on the nurses' well‐being at work (*n* = 271).

Variables	Minimum	Maximum	Mean	SD
Well‐being at work dimensions				
Patients' experience of high‐quality care	1.25	5.00	4.00	0.57
Assistance and support among nurses	1.56	5.00	4.20	0.63
Nurses' togetherness and collaboration	1.10	5.00	4.01	0.78
Satisfying practical organization of work	1.00	5.00	3.30	0.97
Challenging and meaningful work	1.00	5.00	3.66	0.79
Freedom to express diverse feelings in work community	1.00	5.00	3.59	0.98
Well‐conducted everyday nursing	1.00	5.00	3.95	0.78
Status related to the work itself	1.00	5.00	3.69	0.85
Fair and supportive leadership	1.00	5.00	3.63	1.06
Opportunities for professional development	1.00	5.00	3.66	1.03
Fluent communication with other professionals	1.00	5.00	3.90	0.84
Being together with colleagues in an informal way	1.00	5.00	3.37	1.05
Wellbeing at work mean score^a^	1.60	5.00	3.81	0.68
Wellbeing at work overall score^b^	107.00	335.00	255.08	45.24

^a^ Possible score range = 1–5.

^b^ Possible score range = 67–335.

The multiple linear regression model was statistically significant explaining approximately 28.8% of the variance of the nurses' well‐being at work (*F*[18, 252] = 7.08, *p* < 0.001). In Table 4, nationality, type of hospital, and self‐efficacy were identified as significant factors associated with the nurses' well‐being at work. Nurses with Filipino nationality exhibited significantly higher well‐being at work scores than Saudi nurses (*β* = 22.82, *p* = 0.026, 95% CI = 2.76, 42.88). Nurses who were working in private hospitals reported significantly better well‐being at work than nurses in public hospitals (*β* = 13.76, *p* = 0.023, 95% CI = 1.92, 25.61). For the self‐efficacy, a unit increase in the self‐efficacy score was associated with a 4.53 unit (*p* < 0.001, 95% CI = 3.47, 5.58) increase in the well‐being at work score, implying that having higher levels of self‐efficacy among nurses was associated with higher levels of well‐being at work.

**TABLE 4:** Multiple linear regression model for nurses' well‐being at work (*n* = 271).

Predictor variables	*β*	SE‐*b*	Beta	t	p	95% CI	
Age	0.53	0.74	0.08	0.72	0.474	−0.93	1.99
Gender	−1.36	6.58	−0.01	−0.21	0.837	−14.32	11.61
Marital status	3.98	5.34	0.04	0.75	0.456	−6.53	14.49
Nationality (Ref. group: Saudi)							
Filipino	22.82	10.18	0.25	2.24	0.026*	2.76	42.88
Indian	17.84	10.94	0.18	1.63	0.104	−3.70	39.37
Religion (Ref. group: Islam)							
Christianity	3.87	8.08	0.04	0.48	0.633	−12.05	19.79
Hinduism	12.90	12.95	0.07	1.00	0.320	−12.61	38.41
Education (Ref. group: BSN)							
Diploma in nursing	10.89	8.20	0.08	1.33	0.185	−5.25	27.03
Graduate program	3.78	8.23	0.03	0.46	0.646	−12.43	20.00
Hospital	13.76	6.01	0.14	2.29	0.023*	1.92	25.61
Bed capacity (Ref. group: 100 beds and below)							
101 to 200 beds	0.36	6.17	0.00	0.06	0.953	−11.80	12.52
201 to 300 beds	−4.50	9.86	−0.03	−0.46	0.648	−23.93	14.92
301 beds and above	12.51	8.12	0.10	1.54	0.124	−3.47	28.50
Years of experience	−0.03	0.76	0.00	−0.04	0.970	−1.52	1.46
Provided nursing care to COVID‐19 patients	2.84	5.61	0.03	0.51	0.613	−8.20	13.89
Previous COVID‐19 infection	−2.45	5.93	−0.03	−0.41	0.680	−14.13	9.23
COVID‐19 vaccination status	−2.11	6.76	−0.02	−0.31	0.755	−15.43	11.21
Self‐efficacy score	4.53	0.54	0.45	8.46	<0.001***	3.47	5.58

*Note*: The dependent variable was the overall mean score of the nurses' well‐being at work. *β* is the unstandardized coefficients; SE‐b is the Standard error. *R* ^2^ = 0.336, Adjusted *R* ^2^ = 0.288.

* Significant at 0.05 level;

*** Significant at 0.001 level.

Furthermore, the dimensions of well‐being at work and self‐efficacy were tested for their associations. The analyses revealed that all the 12 dimensions of well‐being at work were positively correlated with the nurses' self‐efficacy. The strength of relationships between the well‐being at work dimensions and self‐efficacy ranged from weak (*r* = 0.29) to moderate (*r* = 0.47; see Table 5).

**TABLE 5:** Results of the correlation test between self‐efficacy and well‐being at work dimensions (*n* = 271).

Well‐being at work dimensions	Self‐efficacy	
	*r*	*p*
Patients' experience of high‐quality care	0.47	<0.001***
Assistance and support among nurses	0.43	<0.001***
Nurses' togetherness and collaboration	0.41	<0.001***
Satisfying practical organization of work	0.38	<0.001***
Challenging and meaningful work	0.43	<0.001***
Freedom to express diverse feelings in work community	0.45	<0.001***
Well‐conducted everyday nursing	0.43	<0.001***
Status related to the work itself	0.44	<0.001***
Fair and supportive leadership	0.29	<0.001***
Opportunities for professional development	0.39	<0.001***
Fluent communication with other professionals	0.41	<0.001***
Being together with colleagues in an informal way	0.33	<0.001***

*** Significant at 0.001.

### 3.2 Qualitative results

This part of the study explores the nurses' well‐being during the pandemic. The participants identified four important themes in understanding nurses' well‐being at the workplace.

### 3.3 Safe work environment

During the pandemic, the nurses' workplace was considered safe because changes in the hospital setup were done. Patients were properly screened or test for COVID‐19 before admission to a specific ward assignment. Patients were categorized as COVID‐19 suspected and non‐COVID cases.

“Many changes happened in our hospital setup. Like in our hospital, COVID patients were separated from non‐COVID patients, and the entrance of suspected COVID was separated. Also, in transporting patient, we have separate way and elevator that being use”. (Nurse 4)

The hospital ensured that personal protective equipment needed by the nurses is sufficient, and fitness test for N95 was conducted to ensure the effectiveness of the equipment.

“Supply of PPE is not a problem our workplace, and also when the pandemic start Fit test for N95 was done for all staff nurses”. (Nurse 21)

Nurse feel safe because they are being monitored at work. Their workplace has guidelines and policies for nurses exposed to and with symptoms of COVID‐19.

“We are fortunate in our workplace because if the staff was exposed and developed symptoms, necessary actions were taken by putting the staff in isolation and necessary support were provided”. (Nurse 8)

### 3.4 Ensuring staff nurses' health

The health of the nurses was the priority during the pandemic. Nurses were so overwhelmed by the care being provided by their hospitals to patients. Nurses mentioned that the hospital distributed vitamins and vaccines for influenza and pneumonia to ensure that they would not experience respiratory problems.

“During the pandemic, they ensured that we are healthy. The hospital distributed vitamins and we received influenza vaccine”. (Nurse 2)

Participants recognized the management's effort to ensure that they were healthy because it propelled them to do their work effectively.

“A healthy state of well‐being will make you more competent as a nurse. It is the key for you to provide service above self, which is our main role as a nurse”. (Nurse 21)

To ensure nurses' safety, they are restricted from social gatherings and warned to avoid going to public places. This measure may sound punitive, but it is for their protection.

“For a time, we were not allowed to go out, and all our activities were restricted, but it is okay because it is for our safety and our patient. Especially since I work in the non‐COVID unit”. (Nurse 20)

### 3.5 Leadership support

In the first wave of the pandemic, the nurses described their work conditions as exhausting because of the myriad of challenges that they encountered. There is an issue with implementing the protocol and guidelines in place. They also commented that their leaders and managers were adjusting to the changes in the protocols and guidelines and to new policies. This period of adjustment affected the implementation of the protocols, guidelines, and policies, which led to disorganization. However, the participants expressed that they felt the support that the nursing management was providing to them during those challenging times.

“For a while, things were not organized even though there were clear guidelines, yet in practice, it is different. There is no qualified manager with clear and effective decisions. There was a lack of effective leadership, but our leader is so supportive which gave the feeling that you are not alone and do what you think is right”. (COVID‐RN12)

"Although I work in a private hospital, we admit suspected cases. During this pandemic, we felt the support of our nurse supervisors and administration because training was conducted to ensure that we are protected, and we are safe while providing care to our patients”. (Nurse 19)

Although most mentioned that the administration was supportive and ensured that their professional concerns, such as training on COVID‐19 patients and prospective equipment are sufficient.

“The hospital organization was very supportive during this time of the pandemic. Supplies were provided, especially the PPE, which as absolutely and supremely important. The ever‐changing protocols and guidelines were cascaded to us, and they trained us for any changes. If the staff were exposed and developed symptoms, necessary actions were taken by putting the staff in isolation, and necessary support was provided”. (Nurse 8)

### 3.6 Solidarity in the workplace

Solidarity at work is one of the positive effects of COVID‐19. The participants mentioned that everyone supported each other and ensured that everyone is safe from COVID‐19.

“During the pandemic, the staff working relationship in our hospital was good. Each became each support system”. (Nurse 5)

Solidary is also evident in the good working relationship during the COVID‐19 crisis.

“I have a workmate who does not talk to me for no apparent reason, but during this pandemic, we became close, and she became my buddy in providing patient care”. (Nurse 3)

It was also noted that everyone was very supportive. Teamwork was present, and everyone helped one another and showed support for each other in adjusting and adapting to the nature of care of COVID‐19 patients. The participants felt comfortable providing COVID‐19 patients with care although they were at risk of the disease.

“In the beginning, it was exhausting and stressful because we are facing a new contagious disease. But as we get used to managing the patients, the workload became manageable because of the help of my colleagues”. (Nurse 5)

“The fear that this pandemic brought to us frontliners, was unspeakable. because this virus is new, there were frequent changes in guidelines and protocols, and we were doubtful if this specific protocol would work. But as the days went on, protocols were being established, and we got used to this virus, its nature, and management, and we are getting our feet on the situation. But still, when there are suspected and confirmed patients, fear attacks but not that much, unlike before. We all overcome this situation because of the help we received from our colleagues”. (Nurse 4)

As mentioned by the participant, the spirit of solidarity is evident when a colleague is infected with COVID‐19. Everyone is willing to extend their work hours and work extra hours or days or overtime to cover the work of infected nurses.

“I work extra hours, and I have much overtime because some of our colleagues are infected with COVID‐19. Understaffing is one of the problems, but we need to do extra hours or overtime because it is also a way of helping our hospital and our colleagues. Although it is so exhausting and fulfilling in another way knowing that you are healthy and not having the COVID‐19”. (Nurse 5)

Adjustment to work demands and learning to adapt to the situation during the pandemic is somewhat easy for the participants because of solidarity at work. The participant mentioned that their colleagues are very understanding, more caring, and compassionate at work. They also mentioned that they need to comfort each other because it is their way of uplifting each other.

“We are still adjusting so far, and I could say that this pandemic has helped us become more patient and understanding in all sorts of circumstances. When I am at work, I make sure that my colleague is okay. I always ask him to rest to breathe, especially because we use PPE during our duty. I also received the same concern from my colleagues”. (Nurse 20)

“During the pandemic, the staff working relationship in our hospital was good. Each became each other's support system.” (Nurse 7)

Despite the problem and issues with wearing personal protective equipment, such as having difficulty breathing properly, having difficulty moving properly, and being uncomfortable while working, the participants mentioned that their passion for caring was strengthened. They set aside their fear of being infected and problems brought about by PPE use and ensured that their patients received quality care. They make sure that they live up to their Hippocratic oath of providing quality care regardless of any situation and health problem of the patients.

“I think and firmly believe that we still live by our oath. I am just doing the best that I can to help our patients, living one day at a time and then moving on. Once I started my duty, I knew it was another day that needed my full concentration and assertiveness, trying to leave behind any distractions, may they be emotional burdens or fear, and start my work to help my patients and support my co‐staff. If fear crawls in, my colleagues serve as my support. Then after the duty, I need to move forward and leave things behind in the unit”. (Nurse 19)

## 4 DISCUSSION

The study described and explored nurses' self‐efficacy and well‐being at work amid the COVID‐19 pandemic. The study shows that self‐efficacy of nurses is high amid the pandemic. COVID‐19 is a new disease with no specific management identified during its early stage (Balay‐odao et al., 2021). Despite these adversities, nurses were able to adjust and adapt to these uncertainties (Cruz et al., 2021). Awareness of issues and difficulties helps nurses overcome their problems. Moreover, as noted in the qualitative result, collaboration and teamwork at the nurses' workplace help them adapt and adjust to issues and difficulties amid the pandemic. This finding was also highlighted in the previous studies that nurses perceived teamwork assistance and colleagues as positive aspects of their work and workplace (Balay‐odao et al., 2022; Feldhaus et al., 2019). Thus, self‐efficacy could be associated with teamwork and collaboration of nurses amid the pandemic.

Nurses who work in a private hospital have higher self‐efficacy than those working in a government hospital. This finding could be associated with the nature of the work of nurses amid the pandemic. Abo‐Ali et al. (2021) mentioned that nurses' self‐efficacy is associated with their workplace, nature of work, and type of patient. A previous study in Kenya (Tsuei et al., 2017) and Saudi Arabia (Al‐Ruwaili et al., 2018) shows that type of work and patient affect the self‐efficacy of a person. Most COVID‐19 patients are admitted to government hospitals, and thus, nurses in these hospitals need more adjustment to the nature of work and encounter problems, such as a fear of being infected. Meanwhile, nurses working in private hospitals have fewer COVID‐19 patients under their care, which allows them to have better mental well‐being. Milam et al. (2019) stated that better mental well‐being predicts improved self‐efficacy. This notion could be the reason for the findings of this study.

Self‐efficacy was also found to be a significant predictor of workplace well‐being. The present finding confirms that of Kumar Pradhan et al. (2020), who identified a significant association between self‐efficacy and workplace well‐being. Self‐efficacy is essential to how an individual feels, thinks, performs, comprehends, and changes behaviors and attitudes, enhancing physical and mental welfare (Bandura, 2010). Thus, a person with high self‐efficacy exerts effort to improve interpersonal and organizational systems to have healthy relationships (Ng & Lovibond, 2020). This notion could be associated with the study's findings of the importance of organizational support, leadership support, and solidarity in the nurses' workplace. Similarly, a study conducted among employees in the US and UK shows that high self‐efficacy has a significant influence on relations with others and organization management (Wang et al., 2021).

### 4.1 Limitations of the study

The sample in the quantitative part of the study was obtained by using convenience sampling, which may present issues regarding the generalizability of the findings. The survey was conducted only in the Riyadh region because of some feasibility issues, which excluded other parts of the country. In the qualitative part of the study, the interviews were conducted online because of the restrictions during the pandemic. Hence, it was difficult to consider the non‐verbal cues during the interviews. Nonetheless, the mixed methods design of the study serves as the strength of the study because the qualitative part supports the limitations of the quantitative part.

## 5 CONCLUSIONS

This mixed‐methods study explored nurses' self‐efficacy and well‐being at work during the COVID‐19 pandemic. Overall, the nurses in this study exhibited a high level of self‐efficacy and moderate well‐being at work. The study showed that the nurses' level of self‐efficacy varied between public and private hospital nurses, with the latter reporting higher levels than the former. Being a Filipino, working in a private hospital, and having higher levels of self‐efficacy were linked with better well‐being at work among the nurses. The study further identified important themes in understanding well‐being at work and self‐efficacy during the pandemic, namely having a safe working environment, ensuring staff nurses' health, having leadership support, and having solidarity in the workplace. Thus, the study provides evidence that several factors, such as self‐efficacy, type of hospital, nationality, safe working environment, ensured health, leadership support and solidarity at work play a crucial role in ensuring the nurses' well‐being at work during health crises, such as the pandemic.

### 5.1 Relevance to clinical practice

This study provided rich information on the self‐efficacy and well‐being at work among nurses during health crises, such as the COVID‐19 pandemic, based on its quantitative and qualitative findings. The findings of the study may be used by nurse leaders in developing interventions to ensure that nurses enjoy the highest level of well‐being when working in a clinical setting. The factors identified in the quantitative and qualitative parts of the study should be considered in developing such interventions. For instance, well‐being interventions should focus on all nurses regardless of nationality. Public hospitals should also exert efforts in improving their nurses' well‐being at work. Hospital managers should ensure that the working environment of nurses is a safe place to practice for all healthcare providers. Moreover, hospital managers should guarantee that the health of the nurses and other healthcare providers is promoted, advocated, and guaranteed. Leadership support for nurses is also critical during challenging situations to guarantee the well‐being of nurses. Meanwhile, hospital managers should develop policies that create a culture of solidarity among peers and colleagues in clinical settings. Finally, the findings support the effects of self‐efficacy on the nurses' well‐being. Therefore, hospital managers should support the development of self‐efficacy among nurses by providing continuous development programs and administrative support. It is also important for nurses to constantly discover ways to improve and sustain their self‐efficacy at their work.

## AUTHOR CONTRIBUTIONS

**Nahed Alquwez:** Study conception and design, Data collection, Data analysis and interpretation, Drafting of the article, and Critical revision of the article.

## FUNDING INFORMATION

The author extends his appreciation to the Deputyship for Research & Innovation, Ministry of Education in Saudi Arabia for funding this research work through the project number (IFP2021‐003).

## CONFLICT OF INTEREST STATEMENT

No conflict of interest.

## ETHICAL CONSIDERATIONS

The Research Ethics Standing Committee at Shaqra University approved the study's proposal (Ethics Approval No. ERC_SU_2021008). Electronic informed consent was collected from the respondents to signify their voluntary participation. For the qualitative part of the study, additional informed consent was sought from the respondents. Additional information, such as recording the interview, was included in the consent. The information on the study, as well as the rights of the participants and the potential risks and benefits, were thoroughly discussed with each potential respondent during the recruitment. The investigator's email address was provided in the online survey information section to allow the participants to ask any questions or clarifications about the study. The respondents' privacy and data confidentiality were protected following IRB policy. No identifying information was gathered from the respondents. The risk of undue influence or potential coercion is minimal because of the use of the online survey method for data collection. The recordings of the interviews will be discarded following the policy of the IRB.

## IRB STATEMENT

The protocol of this study was approved by the Research Ethics Standing Committee at Shaqra University (Ethics Approval No. ERC_SU_2021008).

## Supporting information


Supplementary file 1.

Supplementary file 2.

## Data Availability

Data are available from the corresponding author upon reasonable request.
