# Supplementary material for: Nurses' self‐efficacy and well‐being at work amid the COVID‐19 pandemic: A mixed‐methods study
Source: Nurs Open. 2023 Apr 7;10(8):5165–76. doi: 10.1002/nop2.1752 (PMC10333830; doi:10.1002/nop2.1752)
Supplement: Supplementary file 2 [file NOP2-10-5165-s002.docx]

**Supplementary file 2**

**Profile of the participants in the qualitative part of the study**

| **Participant** | **Age** | **Gender** | **Years of experience** | **Nationality** | **Highest educational attainment** |
| --- | --- | --- | --- | --- | --- |
| Nurse 1 | 29 | Male | 6 | Filipino | BSN |
| Nurse 2 | 35 | Male | 8 | Filipino | Masters |
| Nurse 3 | 25 | Female | 1 | Saudi | BSN |
| Nurse 4 | 35 | Male | 13 | Filipino | Masters |
| Nurse 5 | 35 | Female | 13 | Saudi | BSN |
| Nurse 6 | 35 | Male | 12 | Filipino | BSN |
| Nurse 7 | 27 | Male | 5 | Saudi | BSN |
| Nurse 8 | 29 | Female | 5 | Saudi | BSN |
| Nurse 9 | 29 | Male | 8 | Filipino | Masters |
| Nurse 10 | 35 | Male | 13 | Saudi | Diploma/  Associate in Nursing |
| Nurse 11 | 33 | Male | 10 | Filipino | BSN |
| Nurse 12 | 32 | Male | 11 | Filipino | BSN |
| Nurse 13 | 32 | Female | 7 | Filipino | BSN |
| Nurse 14 | 32 | Male | 10 | Filipino | BSN |
| Nurse 15 | 29 | Female | 7 | Filipino | BSN |
| Nurse 16 | 31 | Male | 8 | Saudi | BSN |
| Nurse 17 | 33 | Female | 10 | Filipino | BSN |
| Nurse 18 | 28 | Male | 6 | Filipino | BSN |
| Nurse 19 | 31 | Female | 8 | Filipino | BSN |
| Nurse 20 | 28 | Male | 7 | Filipino | BSN |
| Nurse 21 | 29 | Male | 6 | Filipino | BSN |
